# cAMP Response Element Binding Protein Expression in the Hippocampus of Rhesus Macaques with Chronic Ephedrine Addiction

**DOI:** 10.1155/2017/1931204

**Published:** 2017-10-18

**Authors:** Zongbo Sun, Ye Ma, Shouxing Duan, Lei Xie, Junyao Lv, Jinzhuang Huang, Zhirong Lin, Ruiwei Guo, Shuhua Ma

**Affiliations:** ^1^Department of Radiology, The First Affiliated Hospital of Shantou University Medical College, No. 57 Changping Road, Shantou, Guangdong 515041, China; ^2^Guangdong Key Laboratory of Medical Molecular Imaging, No. 57 Changping Road, Shantou, Guangdong 515041, China; ^3^Department of Linguistics & Languages, Michigan State University, East Lansing, MI 48824, USA; ^4^Department of Pediatric Surgery, The Second Affiliated Hospital of Shantou University Medical College, No. 69 Dongxiabei Road, Shantou, Guangdong 515041, China; ^5^Department of Forensic Medicine, Shantou University Medical College, No. 22 Xinling Road, Shantou, Guangdong 515041, China

## Abstract

**Background:**

Drug addiction is classified as a chronic relapse nature brain disease with complicated neurobiology mechanisms. There are an increasing number of researchers that are investigating the possible mechanisms for solving the thorny problem.

**Methods:**

The model of chronic addiction of rhesus monkey ephedrine was established, where changes in body weight and behavior were monitored. The expression of cAMP response element binding protein (CREB) in the hippocampus of rhesus monkeys was identified by real-time PCR and Western blot.

**Results:**

We were successful in establishing the chronic ephedrine addiction model in the rhesus macaques. They exhibited changes in body weight and behavior. Immunofluorescence showed that CREB was expressed in the nucleus of the hippocampus, and the expression of CREB mRNA and protein in the hippocampus were increased by real-time PCR and Western blot. The CREB positive expression in the hippocampus of the modeling group was significantly higher than in the control group.

**Conclusions:**

The changes of body weight and behavior of the rhesus monkeys after ephedrine chronic addiction were significant. The changes of CREB in the hippocampus of rhesus macaques with ephedrine chronic addiction are important molecular mechanisms, and the upregulation of CREB may be involved in the physiological pathology and behavior process in individuals with chronic ephedrine addiction.

## 1. Introduction

Drug addiction has increased worldwide and poses a serious threat to people's lives and health [1]. Ephedrine is a new-generation drug that has a direct, injurious effect on the nervous system. Current studies on the mechanism of nerve injury in chronic ephedrine addiction have gained notoriety, although the findings remain unclear. The hippocampus is an important brain region that is involved in drug addiction, cue learning, and memory processes. It plays a “bridging” role in the brain stress system through the locus coeruleus-adrenergic system, the hippocampus-ventral tegmental area loop, and the interactions between the hippocampus and prefrontal cortex [2, 3]. The essence of drug addiction is a drug-induced gene expression and neurological synaptic plasticity changes based on pathological memory [4, 5].

Ephedrine has spread to many parts of the world, where it is gradually replacing cocaine, heroin, opium, marijuana, and other drugs to become the world's most harmful and widespread abused drug [6, 7]. Ephedrine is the main raw material used in the synthesis of amphetamines. These drugs can either directly or indirectly act as an agonist to adrenergic receptors, where both *α* and *β* receptors are excited. Ephedrine can excite the cerebral cortex and subcortical center from within the central nervous system, resulting in mental excitement, insomnia, anxiety, and tremors [8, 9]. Chronic amphetamine poisoning in the nerve tissue has a direct impact on the characteristics of the use of such drug addicts to chronic poisoning majority. Therefore, it is important to study the mechanism of nerve injury within chronic ephedrine addiction.

cAMP response element binding protein (CREB) is one of the most important molecular mechanisms that are closely related to addiction memory, where it plays a role of transcriptional regulator in the drug addiction process. Its activation facilitates the cAMP pathway upregulation, which is a type of drug addiction. CREB plays an important role in both the physical and the psychiatric dependence of opioids and central excitatory drugs, particularly within the dopamine receptor-mediated molecular transducers within nuclear signaling. Long-term chronic drug addiction can upregulate the cAMP-PKA-CREB pathway, which leads to abnormal memory formation. Changes of the cAMP-PKA-CREB pathways are key molecular mechanisms of addiction memory [10]. However, since the CREB itself may have gene polymorphism, the role of the target and the specific effects of the different drugs are not analogous. The current study reports on the CREB mechanism of expression in nonhuman primates within ephedrine addiction.

## 2. Materials and Methods

### 2.1. Animals

6 healthy male rhesus macaques were purchased from Guangzhou Xusheng Biological Technology Co., Ltd. Each rhesus monkey had a Guangdong Province experimental animal quality certification. The monkeys were acclimated to the experimental conditions (12 h light dark cycle, 24°C) for 2 weeks. The monkeys had good nutrition, normal mental state, no neurological history, and no history of drug use. The Shantou University Medical College Animal Experimental Ethics Committee approved the use of rhesus monkeys for experimental purposes. The rhesus monkeys were randomly divided into two groups: model group and normal group. The initial basic characteristics are presented in Table 1.

### 2.2. Drug

Ephedrine was purchased from National Institutes for Food and Drug Control (CHN) (number 171241-2011007) and dissolved in normal saline for administration. Drugs were freshly prepared immediately before use.

### 2.3. Establishment of Rhesus Macaques as a Model of Chronic Ephedrine Addiction

Ephedrine injections were administered to the 3 rhesus macaques in the modeling group, starting with a dose of 0.4 mg/kg/d (saline diluent, intramuscular injection) [11]. An intragluteal injection of ephedrine was administered once a day (at 10:00 AM) for three consecutive days and then withheld for one day. The dose was doubled in the second week and doubled again in the third week, until it was maintained at from the beginning of the fourth week throughout the end of the eighth week. The 3 rhesus macaques in the control group were injected with saline at the same dose and time as those in the modeling group.

### 2.4. Rhesus Monkey Weight and Behavioral Observation

All rhesus macaques were weighed weekly, including one week before and after the model establishment during the drug administration phase. Three experimenters with previous professional training independently performed behavioral observation of the rhesus macaques. Addiction behaviors of rhesus macaques, including irritability, piloerection, and runny nose, were observed by using the uniform behavioral scoring criteria [12], three times daily at 8:00 AM, 2:00 PM, and 8:00 PM; observations were performed at least every 2 h. When there were differences in scoring results, the three individuals either discussed and rectified these differences or the animal was observed again to consolidate the results. The average values of the behavioral scores of the rhesus macaques that were recorded twice in the same day were recorded as the behavioral score of that day, according to the observation and measurement results obtained from the three observers. There were four different grades assigned (mild, moderate, severe, and extremely severe) according to the behavioral symptoms of rhesus monkeys during addiction.

### 2.5. Specimen Extraction

The rhesus macaques were euthanized by air embolism. After cutting the skull, the brain was completely removed and the hippocampus was separated. The hippocampus was dissected, where part of the specimen was kept at −80°C and the remaining was put in 10% formalin for further analysis. The specimens were fixed by 10% formalin for 24 hours, embedded in paraffin, and were then sliced in 4 *μ*m thick sagittal continuous slices.

### 2.6. Immunofluorescence for CREB

The samples were dewaxed, antigen repaired, 0.5% Tritonx-100 (AMRESCO, USA) rupture, Goat serum was closed, Plus primary antibody 4°C overnight (1 : 1000 mouse anti-monkey CREB polyclonal antibody (LifeSpan BioSciences, USA)), PBS rinse plus secondary antibody (Alexa Fluor 488 labeled goat anti-mouse IgG (H + L) (abcam, UK), (CREB, green)), and visualized by conventional immunofluorescence with a fluorescence microscope.

### 2.7. Real-Time Reverse Transcriptase-Polymerase Chain Reaction (RT-PCR)

The frozen specimens were added to Trizol (Invitrogen, USA) after grinding. Total RNA was extracted using the one-step method according to the Trizol kit instructions. The concentration and purity of RNA were determined using a NanoDrop® ND-2000 (Thermo Scientific). cDNA reverse transcription was performed using the PrimeScript™ RT reagent Kit with gDNA Eraser (RR047B, TaKaRa, Japan). GAPDH was used as an internal reference protein. The primer sequences and the amplified fragment length are shown in Table 2. PCR conditions were set at 95°C for 30 s and repeated 40 times PCR cycles (95°C, 5 s; 60°C, 40 s (collected fluorescence)). The relative value of the mRNA expression of CREB genes was calculated by comparing the cycle thresholds (CTs) of the target gene with the housekeeping gene (GAPDH) using the 2^−ΔΔct^ method.

### 2.8. Protein Expression Determination Using Western Blot Analysis

The protein was extracted and analyzed via sodium dodecyl sulfate polyacrylamide gel electrophoresis. The gel was removed and the target band was cut, rinsed with distilled water, and transferred onto a polyvinylidene fluoride membrane with a constant 100-mA current. The membrane was blocked using nonfat milk powder and incubated with rabbit anti-monkey CREB primary antibody (1 : 500, LSBio) at 4°C overnight. The membrane was incubated the following day with the secondary antibody (horseradish peroxidase-linked goat anti-rabbit, 1 : 1000, ZSGB-BIO) at room temperature for 1 h. The chemiluminescence reaction, its development, and its fixing were performed. The film was either scanned or photographed, where the optical densities of the target bands were analyzed.

### 2.9. Statistical Analysis

All data is expressed as the mean ± standard deviation. Statistical analyses were performed using SPSS 20.0 software. One-way analysis of variance was performed for comparison and *P* < 0.05 indicated statistical significance.

## 3. Results

### 3.1. The Change in Body Weight of the Rhesus Macaques

**Figure 1:**
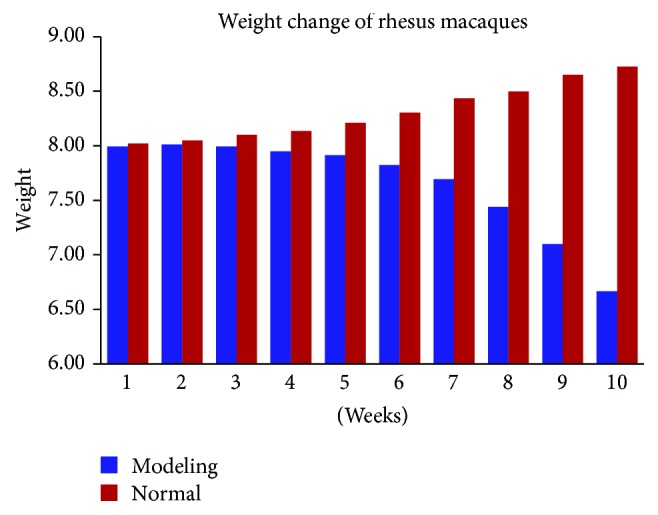
The body weight of rhesus macaques in the modeling group gradually decreased, but it rose in the normal group.

The body weight of rhesus macaques in the modeling group gradually decreased with ephedrine administration. By the third week, the weight changes were significantly lower than before the (*P* < 0.05). However, there was no change in body weight of the rhesus macaques in the normal control group during saline administration (*P* > 0.05). The body weight of rhesus macaques in the modeling group after the intervention was significantly different from that in the control group (*P* < 0.05) (Table 3, Figure 1).

### 3.2. Behavioral Change in Rhesus Macaques

**Figure 2:**
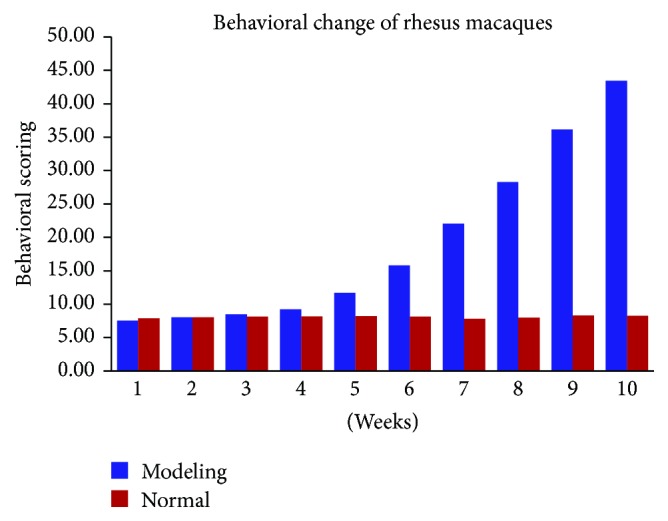
The presence of abnormal behaviors in the rhesus macaques in the modeling group increased after ephedrine administration. There were no obvious changes in the normal group.

The presence of abnormal behaviors in the modeling group increased after ephedrine administration. Student's* t*-test showed that the behavioral score of rhesus macaques after the fourth week was significantly higher than before ephedrine administration in the modeling group and the control group (*P* < 0.01). Behavioral changes were especially obvious by the end of the eighth week, after the completion of modeling. The main manifestations that were present included persistent vertical exploration and climbing. These behaviors gradually disappeared with time and the rhesus macaques exhibited behaviors such as being tired, curled up, listless, and sleepy. They were quick to show a jump-and-attack action, even when given the slightest stimulation. According to the behavioral scoring criteria, differences in the behavioral change of the rhesus macaques in the modeling group were statistically significant before and after intervention when compared to the normal control group (*P* < 0.05). The behavioral change of macaques in the normal control group did not significantly differ before or after the intervention (*P* > 0.05) (Table 4; Figure 2).

### 3.3. Immunofluorescence Analysis of CREB

**Figure 3:**
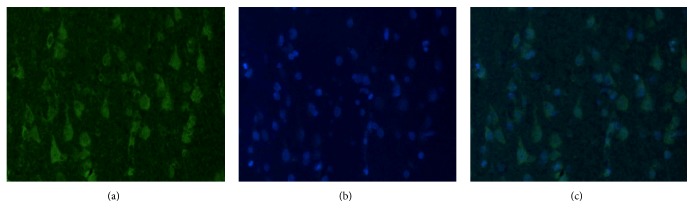
CREB was expressed in the cell nucleus by immunofluorescence. ((a) CREB ×200; (b) DAPI ×200; (c) CREB & DAPI ×200).

The mouse anti-monkey CREB polyclonal antibody was used as the primary antibody, the Alexa Fluor 488 goat anti-mouse fluorescent was used as the secondary antibody for indirect immunofluorescence labeling, and then DAPI staining solution was used for nuclear staining. The Alexa Fluor 488 was stained with green protein (Figure 3(a)), and the nuclei of DAPI blue fluorescence were stained with blue fluorescence (Figure 3(b)). The results showed that the cells in the hippocampus could be specifically labeled by polyclonal antibody against human monkey CREB, which proved the expression of CERB in hippocampal nucleus (Figure 3(c)).

### 3.4. Effect of Ephedrine Treatment on Expression of CREB mRNA

**Figure 4:**
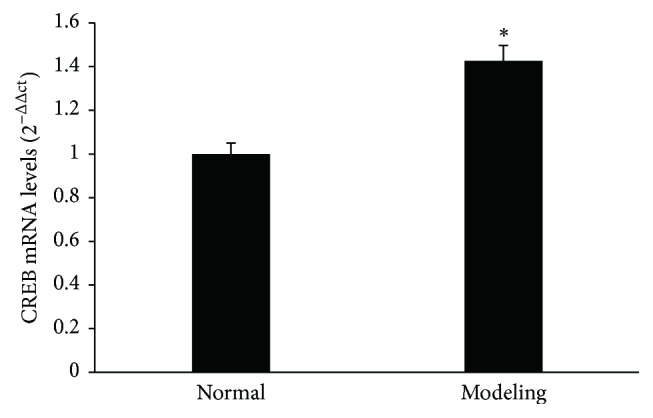
RT-PCR analysis of CREB mRNA levels in hippocampus. Columns indicate mean ± SD, ^*∗*^*P* < 0.05 versus respective control group.

The RT-PCR analysis of hippocampus CREB mRNA levels was significantly higher in the modeling group than in the normal control group (*P* < 0.05) (Figure 4).

### 3.5. Hippocampal CREB Protein Expression

**Figure 5:**
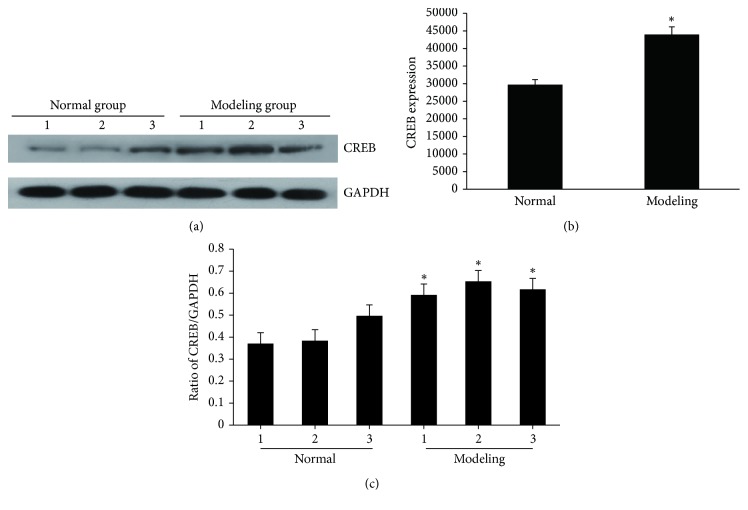
Western blotting analysis of CREB protein levels. (a) The expression of CREB in different individuals, where GAPDH was used as a loading control. (b) Densitometric analysis of Western blot with ratio of CREB/GAPDH. (c) Densitometric analysis of Western blot with CREB expression. ^*∗*^*P* < 0.05 versus control group.

The hippocampal CREB expression was significantly higher in the modeling group than in the normal control group (*P* < 0.05) (Figures 5(a), 5(b), and 5(c)).

## 4. Discussion

Drug addiction is a worldwide medical problem that lacks a quick, effective, and thorough withdrawal treatment method. Numerous studies have attempted to investigate the neurophysiological mechanism of drug addiction in an effort explore new and effective withdrawal methods [13–15]. Ephedrine is one of the most widely used drugs that directly causes harmful nerve injury [8]. Currently, most studies on drug addiction utilize rodent models [16]. However, due to the large anatomical and physiological differences between rodents and humans, the results obtained from these rodent studies lack comparability to their human counterparts. There are many structural, physiological, and pathological similarities among nonhuman primates. Thus, it is of great significance to study these primates to gain an understanding of physiology and pathology in humans. In the present study, rhesus macaques were used to establish the chronic ephedrine addiction model to provide more reliable theoretical evidence for drug addiction.

The hippocampus is an important structure of the limbic system, as well as the advanced regulatory centers of the HPA axis. The HPA axis plays an important role in the regulation of the stress response, which is closely related to drug addiction, learning and memory, and emotion regulation [17]. The mechanism of drug addiction shares many similarities with learning and memory. Some of the main features of addiction are described as another form of memory from a behavioral point of view. CREB is one of the drugs that is repeatedly applied, resulting in gene alteration during the most critical transcription factor expression [18]. The classical learning and memory model suggests that the transcription factor in the CREB plays a key role in the memory process; the role that the CREB plays in the hippocampus is closely related to the formation of long-term memories [19, 20]. In the current study, rhesus macaques with chronic addiction exhibited significant weight loss and behavioral changes. The main manifestations were persistent vertical exploration and climbing. Accordingly, the CREB expression in the hippocampus of rhesus macaques in the modeling group significantly increased, which suggested that the enhanced activity of the CREB in the hippocampus could be involved in the regulation of these behaviors. Thus, upregulated CREB expression could cause weight loss and behavioral changes in the rhesus macaques with chronic addiction.

We hypothesize that the ephedrine led to a change in the CREB activity mediated by the cAMP pathway, where ephedrine was an adrenergic agent that acted on adrenergic alpha and beta-receptors. Adrenergic receptors, a kind of G-protein-coupled receptors (GPCR), mediate the actions of catecholamines. Neurotransmitters within the nervous system (such as ephedrine) travel through the G-protein-coupled receptors to activate the adenylate cyclase, to increase the intracellular cAMP levels, to activate the protein kinases, and to make the CREB Ser-133 phosphorylates that binds specifically to the CREB binding protein (CBP). This resulted in the acetylation of the cAMP response element (CRE) on the promoter while the transcription of the gene was initiated, which then increased CREB expression [21]. The ephedrine upregulation of the CREB could be considered a molecular mechanism of ephedrine addiction; however, its specific molecular mechanisms require further research. Some protein which is a downstream target of CREB (e.g., Nurr1 et al.) [22] will take part in the study and the results will be more sufficient statistically to validate our hypothesis.

## 5. Conclusion 

There was a significant change in both the body weight and behaviors of rhesus macaques with chronic ephedrine addiction. These changes were closely related to the CREB expression in the hippocampus. This suggests that the CREB is involved in physiological processes and pathological behaviors that result from ephedrine addiction. Thus, studying this mechanism is important within underlying chronic ephedrine addiction.

## Figures and Tables

**Table 1:** Basic characteristics of rhesus monkeys.

Parameters	Values (mean ± standard deviation)	
	Model group (*n* = 3)	Normal group (*n* = 3)
Age (years)	5.27 ± 0.26	5.70 ± 0.61
Weight (kg)	7.99 ± 0.06	8.02 ± 0.11

**Table 2:** Primer sequences and amplified fragment length.

Project	Primer sequences	Amplified fragment length
GAPDH	F: 5′ GAAGAGTGGGTGTCGCTGTT 3′	141 bp
	R: 5′ CTGCCGTCTGGAAAAACCT 3′	
CREB	F: 5′ GTGCCAGCCTTTCCTTACAC 3′	109 bp
	R: 5′ CACAAACCCACTGATGAACG 3′	

**Table 3:** Weight change of rhesus macaques ($\overset{-}{x}\pm S$) (unit: kg).

Groups	Weeks									
	(1)	(2)	(3)	(4)	(5)	(6)	(7)	(8)	(9)	(10)
Normal group (*n* = 3)	8.02 ± 0.11	8.05 ± 0.11	8.10 ± 0.10	8.14 ± 0.09	8.21 ± 0.11	8.30 ± 0.03	8.43 ± 0.08	8.50 ± 0.05	8.65 ± 0.04	8.73 ± 0.09
Modeling group (*n* = 3)	7.99 ± 0.06	8.01 ± 0.10	7.99 ± 0.10	7.95 ± 0.08^*∗*^	7.91 ± 0.08^*∗*^	7.82 ± 0.04^#^	7.69 ± 0.02^#^	7.44 ± 0.05^#^	7.10 ± 0.05^#^	6.67 ± 0.05^#^

^*∗*^ *P* < 0.05 versus group of normal. ^#^*P* < 0.01 versus group of normal.

**Table 4:** Behavioral change of rhesus macaques ($\overset{-}{x}\pm S$) (unit: grade).

Groups	Weeks									
	(1)	(2)	(3)	(4)	(5)	(6)	(7)	(8)	(9)	(10)
Normal group (*n* = 3)	7.92 ± 0.72	8.02 ± 0.71	8.15 ± 0.67	8.17 ± 0.68	8.23 ± 0.25	8.11 ± 0.09	7.82 ± 0.19	7.98 ± 0.16	8.31 ± 0.10	8.27 ± 0.43
Modeling group (*n* = 3)	7.55 ± 0.50	8.06 ± 0.20	8.50 ± 0.22	9.24 ± 0.40	11.71 ± 0.76^#^	15.81 ± 1.51^#^	22.05 ± 1.73^#^	28.28 ± 3.86^#^	36.13 ± 3.41^#^	43.45 ± 1.35^#^

^#^ *P* < 0.01 versus group of normal.
